# Capturing the design space of meaningful human control in military systems using repertory grids

**DOI:** 10.3389/fpsyg.2025.1536667

**Published:** 2025-07-16

**Authors:** Jurriaan van Diggelen, Christine Boshuijzen-van Burken, Julian Vince

**Affiliations:** ^1^TNO Defence, Safety and Security, Soesterberg, Netherlands; ^2^UNSW Canberra, Kensington, NSW, Australia; ^3^Defence Science and Technology Group, Melbourne, VIC, Australia

**Keywords:** meaningful human control, responsible AI, repertory grids, AI in defense, autonomous weapon systems

## Abstract

This study explores the design space of meaningful human control (MHC) for military AI systems using the repertory grid technique (RGT) to capture expert perspectives across disciplines. By interviewing twelve experts from fields such as military, engineering, philosophy, and human factors, we identified key constructs related to autonomy, moral sensitivity, destructiveness, and human-machine interaction. The findings reveal both consensus and variation in experts’ understanding of MHC, highlighting challenges in interdisciplinary alignment. The study identifies key variables for MHC and uses them to create a design map that guides system designers in integrating MHC concepts into AI applications. By establishing a shared vocabulary and improved elicitation methods, we aim at facilitating future discussions and research aimed at establishing and maintaining MHC.

## 1 Introduction

The concept of meaningful human control (MHC) has gained prominence in discussions about AI-enabled and autonomous weapon systems. MHC asserts that as AI and autonomous technologies progress, humans should maintain control over moral decisions, including those regarding the use of force. Various organizations, including NATO^1^ and the US Department of Defense,^2^ have framed sustaining MHC as both an ethical and potentially legally binding principle. In 2023, the United Nations Secretary-General and the President of the ICRC called on states to negotiate a legally binding agreement: “*We must act now to preserve human control over the use of force. Human control must be retained in life and death decisions*.” Despite these calls to action, no legally binding principle has been formulated yet, and it remains unclear how to translate MHC into concrete, implementable, and verifiable design requirements, and specifications.

Part of this deficiency is caused by the challenges of establishing an effective multidisciplinary dialog. Military MHC research spans a range of fields, including philosophy, law, military doctrine, human factors, and AI technology. Therefore, establishing common concepts and a shared language that all parties can understand is essential. For instance, while Human Factors specialists may advocate that the operator’s “situational awareness” is crucial for MHC, its significance may be lost on technology developers or policymakers if not effectively communicated and integrated into their frameworks. This research attempts to remedy this shortcoming.

The primary aim of this study is to explore how experts from various disciplines understand the concept of MHC, using specialized semi-structured interviews (i.e., the *repertory grid technique*). Our central research question is: *What are the shared and differing conceptual elements that specialists from diverse fields use to define meaningful human control?* A secondary goal is to propose a method for translating these insights into improved mutual understanding and a practical approach for implementing MHC across domains. Framed as a question: *Can we develop a design framework with actionable requirements that policymakers and system designers can adopt to strengthen MHC in AI and autonomous systems?*

Several methods have been suggested to bridge the gap between disciplines and integrate ideas from experts across various fields. These include unstructured interviews, questionnaires, systems thinking (Bonnema and Broenink, 2016), and the repertory grid technique (RGT) (Hassenzahl and Wessler, 2000). Each method varies in the degree of predetermined structure it imposes on the data acquisition and analysis process, as well as the type of expertise it gathers. For our purposes, we believe the RGT offers the right balance. Repertory grid is a well-established semi-structured interviewing technique that has been proven successful for the extraction of mental models in various domains. It produces data for both qualitative and quantitative analysis. While RGT has been used in design problems outside AI and military contexts (Hassenzahl and Wessler, 2000; Kwak et al., 2014; Tofan et al., 2011), applying it to the issue of responsible military AI design is a novel approach.

Using RGT, we have gathered data from 12 experts in the field of MHC spanning various disciplines such as military, philosophy, human factors, engineering. We approached the experts by stressing that we were interested in a socio-technical systems design perspective. This perspective emphasizes that the designer’s focus extends beyond merely crafting technology; it also involves shaping an environment conducive to human effectiveness and safety, designing seamless human-machine interactions, and ensuring the system’s utilization is restricted to specific contexts, among other considerations. We presented eight different human-AI systems relevant to the MHC debate and employed the RGT to elicit constructs that highlight the differences between them.

We analyzed the data in three phases. In the explorative phase we performed a principal component analysis which showed that some human-AI systems were interpreted as being more similar than others, although the constructs which experts used to point out these similarities were highly different. In the interpretative phase we categorized the similar constructs and explored to which extend the expert’s mental models were different and similar. In the constructive phase we used these insights to create a common vocabulary and demonstrated how it can be used to derive a basic design framework that lays out the different design options that are relevant for MHC.

The paper is organized as follows. The next section provides background on the MHC debate, and connects it to research on multi-disciplinary design, including a description of the background development and application of RGT. Section 3 discusses the method we used for the RGT study. Section 4 discusses analysis of the results following the explorative, interpretative, and constructive phases. Section 5 concludes the paper with reflections on our experiences using RGT for responsible military AI design and proposes directions for future research.

## 2 Background

This section provides background on the MHC debate and the repertory grid method.

### 2.1 Meaningful human control debate

The MHC debate has been ongoing for nearly a decade. Attempts to define, refute, or give meaning to the concept has been ongoing in different disciplines and institutions, such as academic debates and policy documents.

For example, the United Nations Group of Governmental Expert (GGE) on Emerging Technologies in the Area of Lethal Autonomous Weapons Systems states: “*Although there is agreement on the importance of the human element…, [f]urther clarification is needed on the type and degree of human–machine interaction required, including elements of control and judgment*” (UNGGE CCW, 2019). The Responsible AI in the Military (REAIM) Blueprint states that “appropriate levels of human control” needs to be maintained, which includes measures relating to “human judgment and control over the use of force” (REAIM, 2024). States use different terminologies in their policy documents, for example the USA’s Department of Defense Directive 3000.09, Autonomy in Weapon Systems requires that “*[a]utonomous and semi-autonomous weapon systems shall be designed to allow commanders and operators to exercise appropriate levels of human judgment over the use of force*” (US Department of Defense, 2023). The UK DoD states the following about the principle of “*Human Control: when using AI-enabled systems for Defence purposes, the need to understand the appropriate form of human involvement required for any given application or context*” (UK Ministry of Defence, 2022). These latter formulations about the role of humans in relation to AI are provided by military organizations where human control is always *assumed* and closely tied to human responsibility through a clear chain of command, which is visibly or verbally expressed through ranks, directives, or orders, at all levels in the organization. However, while there is some consensus, there is also disagreement (or failure to make progress). This is, as we argue in this paper, because of a lack of common concepts and shared vocabulary that spans the different disciplines. When we look at academic debates, Santoni de Sio and Van den Hoven (2018) gave a philosophical account of MHC and problematized the concept, pointing out the needs and challenges with MHC. Moral grounds for MHC were suggested by Verdiesen et al. (2020), who suggested to rename the concept to Comprehensive Human Oversight.

Kwik (2022) took a legal lens to the uses and applications of the term, and identified five core elements of MHC in policy documents and official statements: awareness, weaponeering, context control, prediction and accountability. Davidovic (2023) summarized the moral and legal purposes of MHC over military-AI as follows: safety and precision, responsibility and accountability, morality and dignity, democratic engagement and consent, and institutional stability. Kwik’s literature-based research and Davidovic’s conceptual work is helpful, however, it lacks a forward looking approach to understanding MHC across disciplines. We analyze MHC by using a social science tool to distill key features of MHC as perceived by experts with a background in different disciplines. Our approach is empirical, rather than based on previous conceptual work on MHC.

Finally, we note that the ongoing MHC debate takes place in the context of responsible military AI, where the question of how humans retain and exercise responsibility in practice has been a central issue. MHC is relevant for safeguarding the traditional *jus in bello* principle of distinction (i.e., discerning between combatants and non-combatants) and indirectly to the principle of proportionality (i.e., ensuring that harm to civilians is not excessive in relation to the anticipated military advantage). These principles, which are formalized in International Humanitarian Law (IHL), must be adhered to during the use of AI in military operations. Although AI-enabled systems may technically be capable of distinguishing between combatants and non-combatants or assessing proportionality, military operations are inherently unpredictable, chaotic, and deceptive which makes it difficult to reliably apply such judgments. Kwik (2022) gave a helpful overview of the salient legal features of autonomous, and AI enabled military systems, such as temporal variables, environmental dynamicity, spatial dimensions. In our empirical method, we systematically investigate some of these variables and how they affect different understanding of MHC across disciplines.

### 2.2 Terminological confusion around AI and MHC

In general, definitions of AI and autonomy in systems are not universally agreed upon (with notable differences in for example the EU AI Act^3^ and the OECD^4^ definition) and this has implications for the MHC discussion, as MHC is closely tied to applications of AI and autonomy. Confusion of the term, and differences in interdisciplinary interpretations, do not help in the debates around autonomy (Burton et al., 2020).

This paper does not aim to provide a new definition of MHC; instead, it seeks to identify common themes in MHC debates across disciplines, pointing to areas of overlap and divergence in how people describe MHC. We investigate how RGT analysis can be used to guide cross-disciplinary interview sessions to come to a set of shared variables. These shared variables can be used as a basis for design guidelines for human-AI systems. This paper provides the basis for a variety of concrete design options to create or ensure MHC, which we call design patterns (van Diggelen et al., 2024b). They have arisen through an empirical data gathering method, namely RGT where we asked people from multiple disciplines what comprised human control in various vignettes. The vignettes depicted socio-technical systems with various types of human control (more on this latter).

### 2.3 RGT as a method to come to design choices

Empirical data gathering on a specific topic can be done in a variety of ways. Unstructured or semi-structured interviews allow the interviewee freedom to come up with new ideas. Data resulting from interviews are often difficult to analyse scientifically. Data that is gathered through survey methods such as Likert scales are easier to analyse through scientific statistical analysis but restrict participants in their freedom to express themselves. RGT provides a middle ground by allowing the interviewee to formulate concepts themselves and uses these concepts to generate data that is statistically analyzable. There is no pre-defined number of participants for an RGT, but typical number of participants may be considered low, for example Bodycott (1997), who investigated conceptions of the “ideal teacher,” interviewed 12 participants, Richter et al. (2022) interviewed 9 participants, whereas Kozikoglu (2017), selected 6 participants in their RGT study.

RGT was first proposed by Kelly (1955) as a tool for creating a theory of personality. It provides a tool for understanding how different people see the world. It is systematic but allows freedom to capture individual mental frameworks that can then be collated and contrasted with other individuals’ mental frameworks. RGT elicits personal “constructs” around a theme or experience. Constructs are not exactly concepts. Constructs represent some form of judgment or evaluation about a topic, formed through a person’s life experience. Constructs in RGT are always scalar and dichotomic: e.g., the concept *hot* can only exist in contrast to the concept *cold*, the concept *weak* can only exist as a contrast to the concept *strong*. “*[I]f we seek to capture an individual’s personal meaning with precision, we need to make the particular contrast explicit, and that is what Repertory Grid Technique is designed to do*” (Bourne and Jankowicz, 2018, boldface by us). A typical RGT process takes three “elements” (concrete exemplars within the topic under investigation), provides these to the participant, and asks for two of them to be paired in contrast with the third. The participant is then asked to formulate how the two elements are similar and what makes these two different from the third. The “how (not) similar,” is basically the construct and this is how two poles of the construct can be elicited. This exercise of asking participants to pair and contrast three constructs is repeated for a number of elements. Either under the guidance of a researcher/facilitator, or by working individually, participants populate a RepGrid, which is a matrix consisting of columns, in which elements are listed, and rows for the constructs (Alexander et al., 2010) (e.g., see Table 1). The elements are specific, concrete examples from the topic under study. Elements are used to help the participants to identify their own “constructs” [cf. Kelly (1955)] or perceptions regarding the particular research topic that is being considered.

**Table 1:** Example of a repertory grid extracted from one of the participants.

		Elements								
Construct	Leftpole (1)	1	2	3	4	5	6	7	8	Rightpole (5)
Moral permissibility	High	1	2	2	5	2	1	3	5	Low
Control moment	Continuous	1	1	5	5	3	3	5	3	Prior
Domain of operation	Physical	1	1	1	1	1	1	5	5	Virtual
Situational awareness	High	5	5	2	1	2	2	5	2	Low
Moral sensitivity	High	5	2	4	1	4	3	3	1	Low
Task load	High	2	1	5	5	1	2	5	3	Low
Context of use	Narrow	3	4	2	5	4	3	1	4	Broad
…	…	…	…	…	…	…	…	…	…	…

RGT continues to be studied and refined, with various adaptations documented by researchers such as Rahman et al. (2022). They categorize the degree in which researchers are involved in construct and element elicitation. In their overview, our research would fall into category (2):

“*(2) Minimum context form (triadic sort method), in which each elicitation entails the selection of three random elements from the full set. Here, participants identify how two elements are similar yet different from the third. Researchers may provide participants with contextual cues to facilitate their attention toward a specific research problem. The researcher repeats the elicitation process until all relevant constructs are identified. Research has shown that in most domains, the required number of triads to elicit constructs from participants is usually 7–10* (Reger, 1990).”

Hadley and Grogan (2022) discussed relative strengths and weaknesses of RGT and noted that the methods is relatively intense for participants in terms of time and cognitive effort. Researchers must furthermore be constantly on the lookout that participants do not make mistakes when rating the constructs, or are not adequately expressing the implicit construct, or provide poor elements. RGT’s greatest shortcoming, according to Fetters and Molina-Azorin (2017), is that it cannot be administered to a large number of participants and works best with individuals rather than with large groups.

RGT has been applied in the context of design by Hassenzahl and Wessler (2000). They tried to distinguish good ideas from bad ideas in parallel design processes. Hassenzahl and Wessler explored the practical value of RGT in gathering design-relevant information on the design space of early artifact prototypes. They found the method particularly useful to capture “*topics, thoughts, and feelings—in short, information— that do not fit into the predetermined structure [of pre-structured approaches, i.e., questionnaires]. This is especially problematic if there is a general lack of knowledge about the topic to be researched*.” (ibid). The lack of general knowledge is precisely the case for MHC, as we have argued before. Open semi-structured interviews can solve the problem of rigidity, but interviews require research effort in terms of transcribing hours of interview data and outcomes depend on the interpretation of the researcher doing the data analysis. RGT makes it possible to understand an individual’s personal (i.e., idiosyncratic) construction of the topic under investigation, which can be artifacts, phenomena, other persons (e.g., members of professional or social groups). Hassenzahl and Wessler found that what is helpful in design, is to distinguish the particularities and disagreements between designers, which is often overlooked in attempts to make design choices based on where the most agreement is Kwak et al. (2014) provide a helpful summary of RGT studies, most notably found in human-computer interaction and information systems. They state that early uses focused on RGT for knowledge elicitation and that later studies captured users’ understanding of digital artifacts through eliciting users’ constructs about those artifacts, or how users understood the usability of six systems they interacted with on a regular basis. They also contrasted the RGT across participants with different national and professional, backgrounds, which resembles the interdisciplinarity of the MHC debates and is therefore a relevant example. Kwak et al. themselves used RGT to elicit knowledge for designing artefacts (shape-changing interfaces) and found the construction of a vocabulary highly important for this niche design-space of shape-changing interfaces.

Tofan et al. (2011) used RGT to capture tacit architectural knowledge of software intensive systems. In contrast to the previous studies discussed above, Tofan et al., used the method to ask participants about existing previous decisions regarding systems, while the other studies introduced participants to a novel artifact. Our approach uses both strands as we ask participants about previously experienced human-machine systems (such as the car) as well as novel or future human-machine systems.

## 3 Methods

Using the RGT as outlined by Hassenzahl and Wessler (2000), we conducted a series of twelve 2-h interviews from March to May 2024. Additional details are provided in the following sections.

### 3.1 Participants

Data was gathered from twelve experts across diverse fields: from philosophy (3), human factors (4), engineering and artificial intelligence (4), and from the military (1), with participants from either Australia or the Netherlands. Each expert had over 10 years of relevant experience and was regarded as a leader in their field. They were well acquainted with the topics raised in the interviews. Participation was entirely voluntary. Each expert signed an informed consent form prior to the interview, and their anonymity was preserved such that their responses could not be traced back to individual participants. The study received approval from DSTG’s Low-Risk Ethics Panel (LREP) under protocol number DSTG-HADS-202403010353. RGT interviews were conducted by all three authors, who first practiced and sat in each other’s practice interviews to identify issues and enhance uniformity in the data gathering process.

### 3.2 Materials

**Figure 1:**
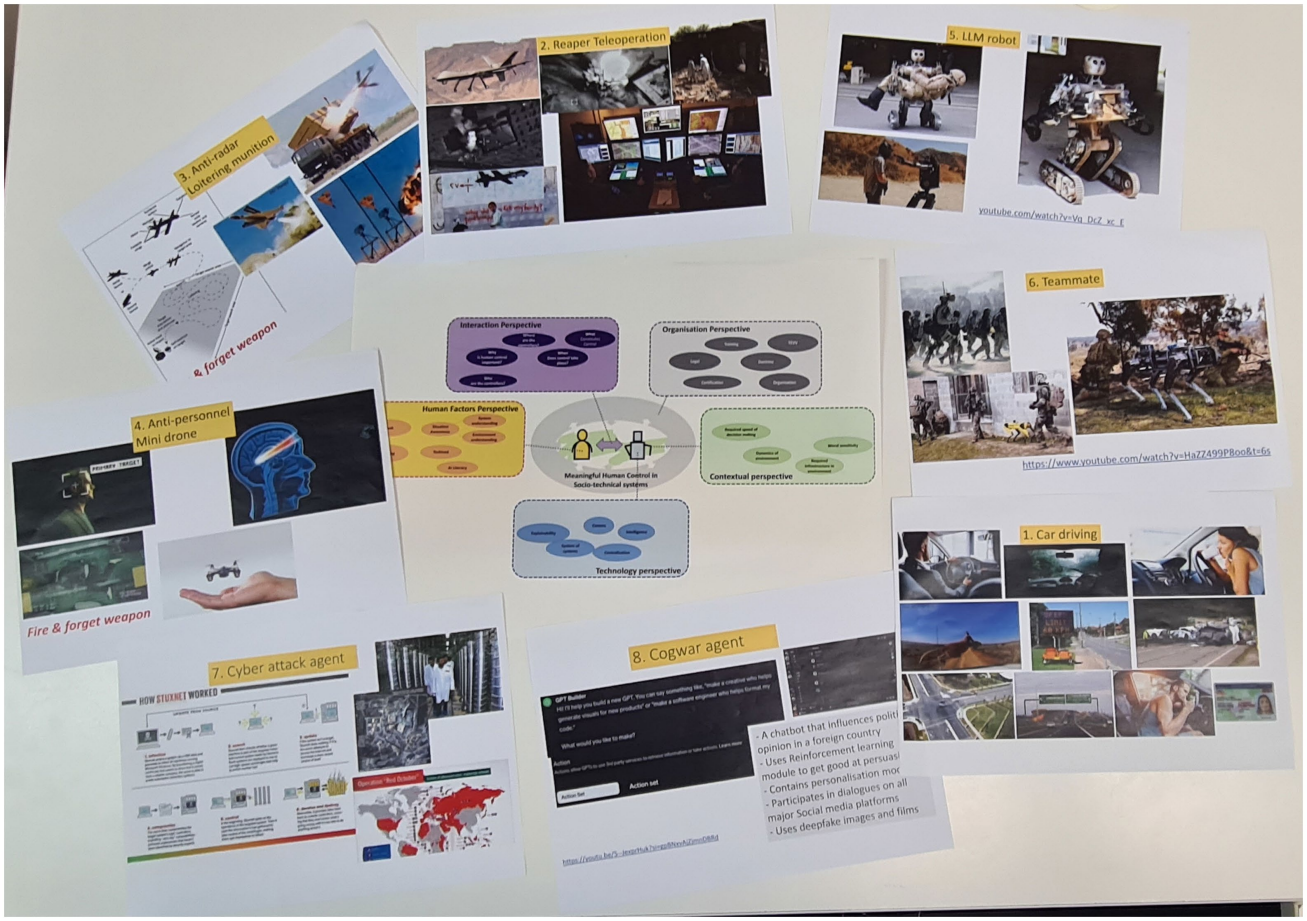
Material presented to the interviewees.

The participants were presented with eight distinct types of (military) human-machine/AI systems, chosen to represent realistic (now or in the future), representative and diverse cases. In RGT terms, these would be called the elements. These use cases were selected based on our experiences in NATO working groups (e.g., HFM-RTG-330) and prior research (e.g., van Diggelen et al., 2024a,b). Each use case was presented on an A4 sheet displaying its name and a series of photos (see Figure 1). The participants were then given a brief oral explanation by the researcher/facilitator of each case using the following text:

#### 3.2.1 Car driving

Driving a car is probably the most familiar example of a human controlling a machine. But controlling a car takes place in a larger system, as illustrated by these examples. Often, changes in the environment cause loss of control, e.g., a kangaroo, or heavy rain. Loss of control can cause deadly accidents. The infrastructure is built to minimize potential accidents; for example there are permanent or temporary speed restrictions, road signs, car-lanes. The driver should be trained and have a license; otherwise it’s illegal to drive. The driver should be capable to drive. Do not drink coffee while driving, and definitely do not drink and drive!

#### 3.2.2 Reaper teleoperation

The reaper drone is controlled by two trained operators: a pilot controlling the aircraft, and a sensor operator who also guides weapons. The operators can view live video footage and other sensor streams, and control the UAV using joysticks, buttons and switches. They operators may be part of a larger team containing an intelligence analyst, mission coordinator, legal advisor. The operators can be thousands of kilometers away from the drone. The drone may be armed with weapons that can have disastrous effects on the ground. Sometimes only the target is destroyed. Sometimes collateral damage causes great suffering for people on the ground.

#### 3.2.3 Anti-radar loitering munition

An anti-radar loitering munition system is a small “suicide drone” armed with sensors and a warhead. Before take-off, the human controller enters the unique radar signature of the target to be attacked by the loitering munition. After take-off, the drone hovers for up to 2 h over an area, scanning for the radar signals. After take-off, there’s no remote communication and the drone can no longer be controlled (hence the name fire&forget weapon). Once it detects the radar, it attacks the target “kamikaze style.” The loitering munitions can be launched from a mobile truck.

#### 3.2.4 Anti-personnel mini drone

This hypothetical system was portrayed in the documentary “slaughterbots”.^5^ It can be considered as a variant of the Anti-radar Loitering munition but much smaller and designed to attack people. Before launch, the human controller uploads an image of the face of the target. After launch, the system flies around and scans people’s faces for a match using it’s deep-learning facial recognition system. If it positively identifies the target, it lethally attacks the person by using a small penetrating explosive bullet on the person’s skull. It’s also a fire and forget weapon.

#### 3.2.5 LLM robot

Please watch the movie on a Large Language Model (LLM) robot.^6^ Suppose we have this type of general-purpose robot which can be controlled using an LLM (such as chatGPT) For example, the user could write a prompt that would direct it to recover soldiers from the battlefield. The user could also prompt it to carry stuff, use sensors, and deploy weapons, etc. The user can prompt anything that comes to his or her mind.

#### 3.2.6 Teammate

This fictional system is even more advanced and acts like a true teammate. Please watch this fragment from the classic science fiction movie “Terminator”.^7^ Note that Arnold Schwarzenegger is a humanoid robot; the child is human. What we saw in the movie was a robot that thinks along with the human, takes initiative, and make agreements about how to cooperate. Although this is science fiction, a serious future vision is that humans will work with machines just as humans work with their human team-members. In military teams, one could imagine dog-shaped, or humanoid robots to become part of the team. The robotic teammates would be subordinated to humans, and behave like lower ranked soldiers.

#### 3.2.7 Cyber-attack agent

This autonomous system acts in cyber domain, inspired by Stuxnet^8^. A team of cyberwarfare specialists released a computer virus which would spread and infect computers worldwide. In most cases, the virus would do nothing and would probably not even be noticed. The virus would activate its malicious code only after detecting a very specific computer (i.e., a centrifuge that can be used to enrich uranium). If the virus failed to infect the nuclear centrifuges, the cyberwarfare specialists could tweak the virus to make it more “contagious.” After the virus was released, the cyberwarfare specialists could not control or retract it.

#### 3.2.8 Cogwar agent

This case is similar to the LLM agent, and is inspired by Tristan Harris’ “alpha persuade” system,^9^ and GPT Builder. It can be directed using LLM prompts (e.g., using chatGPT), to spread false narratives, fake news, framed news, and conspiracy theories on social media. It infiltrates social media communities, participates in dialogs, and delivers a personalized message to the user. It learns to get better at persuasion using a reinforcement learning algorithm that monitors the effects of its actions It uses generative AI for text, images, and movies.

**Figure A1:**
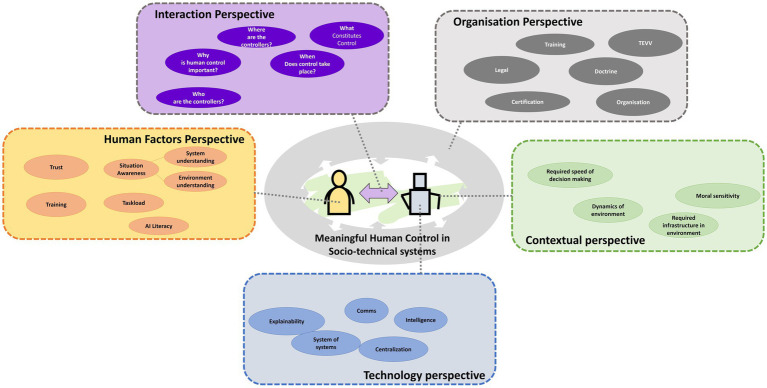
Idea-stimulating materials used during interviews.

Besides these eight sheets with cases, they were presented with a diagram that stresses the socio-technological perspective (see the sheet at the center of Figure 1 and Figure A1). This sheet was not explained in depth but served to encourage the participants to take diverse perspectives: the human factors perspective; the technological perspective; the context perspective; the organizational perspective; the interaction perspective.

### 3.3 Procedure

Each participant was interviewed individually, with about half of the interviews conducted face-to-face and the rest over video calls. Before the interview, participants received the materials described in the previous section. For face-to-face interviews, printed versions of the cases and the socio-technical system diagram were laid out on the table. For online interviews, participants were asked to have the materials readily accessible on their desktop for easy reference.

The interview process comprised three parts: Practice, Elicitation, and Open questions.

#### 3.3.1 Practice

Participants were introduced to the repertory grid method using a simple practice example involving four elements—Cat, Fish, Dog, and Elephant.^10^ Participants were asked to identify a dimension (personal construct) where two elements were similar (inclusive construct pole) and differed from the third (exclusive construct pole). They then labeled each pole to describe the dimension concisely. For example, when presented with the triad <*Cat, Dog, Elephant*>, they might answer that <*Cat, Dog*> are similar and different from <*Elephant*> on the basis of construct <*Pet-suitability*>. The inclusive construct pole could be *Suitable-as-pet,* and the exclusive construct pole would be *Not-suitable-as-pet.* Then, a new triad was presented, and they were asked the same question until no new constructs could be generated. Finally, the participant rated each element on their constructs using a scale from 1 (inclusive pole) to 5 (exclusive pole). This part of the interview lasted approximately 10 min.

#### 3.3.2 Construct and repgrid elicitation

Participants were introduced to eight realistic or hypothetical future military use cases (described in the previous section). After the interviewer read the case explanations aloud, the participant was invited to ask any questions they had about the human-machine systems. Then, the repertory grid procedure began, following the same steps as practiced previously with the Cat, Fish, Dog and Elephant, but now applied to the actual cases. For each case, they were asked “From a perspective of control in a socio-technical system, can you choose two of this triad of elements, which are in some way alike and different from the other one?” This led to a set of constructs with corresponding construct poles. Next to the constructs they had generated themselves, we added one construct to the repertory grid, called “morally permissibility,” with poles *Morally permissible* and *Morally wrong*. They were asked to fill in the repertory grid for all the constructs on all elements. This part of the interview lasted approximately 60 min.

#### 3.3.3 Open questions

Participants were asked open-ended questions, such as:

- Why do you think some elements were morally wrong while others were permissible?
- Do you feel any representative elements for MHC might be missing now or in the future?
- What is your opinion on the interview and the RGT?

This part of the interview lasted approximately 20 min.

### 3.4 Data

An example of the first rows of a repertory grid is presented in Table 1. The repertory grid shows the construct names in the dark green column. In the light green column, the left and right pole values of these constructs is shown. In the yellow cells, the values for each of the elements are shown. The element numbers correspond to the numbering of elements presented in Section 3.2, i.e., 1 Car driving; 2 Reaper teleoperation; 3 Anti-personnel mini drone; etc. The yellow cells show the values that the subjects assigned to the elements, where 1 corresponds to the left-pole value, and 5 corresponds to the right-pole value. For example, the subject that generated the grid in Table 1 assigned the control moment of the Reaper (element 2) as highly *continuous*, and the control moment of the Anti-personnel mini drone (element 3) as *prior*.

The entire interview process led to 12 repertory grids (i.e., one for each participant). The grids were between 10 and 25 rows long. The total number of constructs generated by all participants was 156. Multiplied by the 8 elements, this gave us 1,248 data values (shown in the yellow cells).

## 4 Results

Similar to the three step process outlined by Hassenzahl and Wessler (2000), we analyzed the data in three phases (1) exploration, (2) interpretation, (3) application.

### 4.1 Exploration

A typical method for analyzing repertory grid data is through Principal Component Analysis (PCA). The purpose of Principal Component Analysis (PCA) is to reduce the dimensionality of a dataset by transforming it into a set of orthogonal components that capture the variance of the data. This simplification helps to identify underlying structures in the data, making it easier to analyze and interpret. Using the RGT software package,^11^ we applied PCA to the entire set of repertory grid data. It revealed seven components that explain the variance in the data as follows: 42, 28, 14, 7, 4, 3, and 2%. By plotting the two most significant components on the X and Y axes, we obtain the visualization shown in Figure 1. This figure highlights similar and dissimilar elements. Since the first two principal components account for 70% of the variance, elements that are positioned closer together on the plot indicate that respondents attributed similar characteristics to them. The following clusters can be identified: [*LLM robot, Teammate*], [*Anti-Radar Loitering Munition, Anti-Personnel Mini Drone*], and to a lesser extent [*Cyberattack agent, Cogwar agent*]. By looking at the blue texts that are close to the elements, we can identify which constructs respondents used to characterize that element. For example, the cogwar agent is associated with *Geopolitical, non-predictable outcomes*, and *High-AI-literacy needed*. Note that these constructs are individual responses and do not represent a consensus. This plot is not particularly useful for identifying common constructs, as it mainly shows that respondents used a wide variety of constructs (156 in total), each with distinct meanings, as indicated by their widespread distribution across the plot. What this figure does make clear, however, is that debates on MHC, AI and autonomy are messy due to a lack of coherent meaning across disciplines, as we have argued in the background section of this paper. Identifying common constructs is a key focus of this paper, and we will explore this issue further in the next phase of analysis, which will be detailed in the following section (Section 4.2).

**Figure 2:**
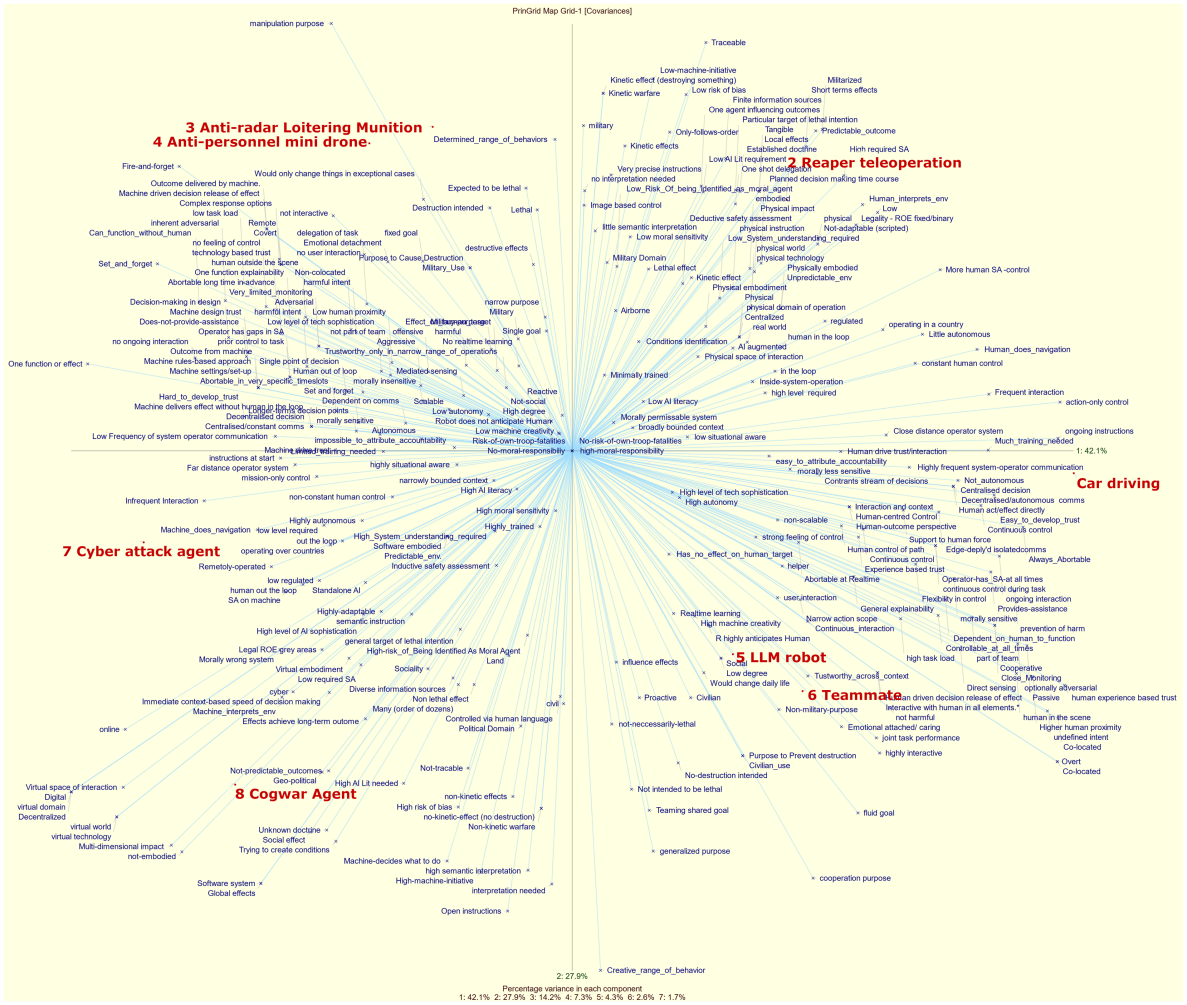
Principal component analysis of aggregated expert data (*n* = 12) on all eight elements.

However, before addressing this topic, we will first analyze the ratings of the one construct that we requested from all participants: *moral permissibility*.

**Figure 3:**
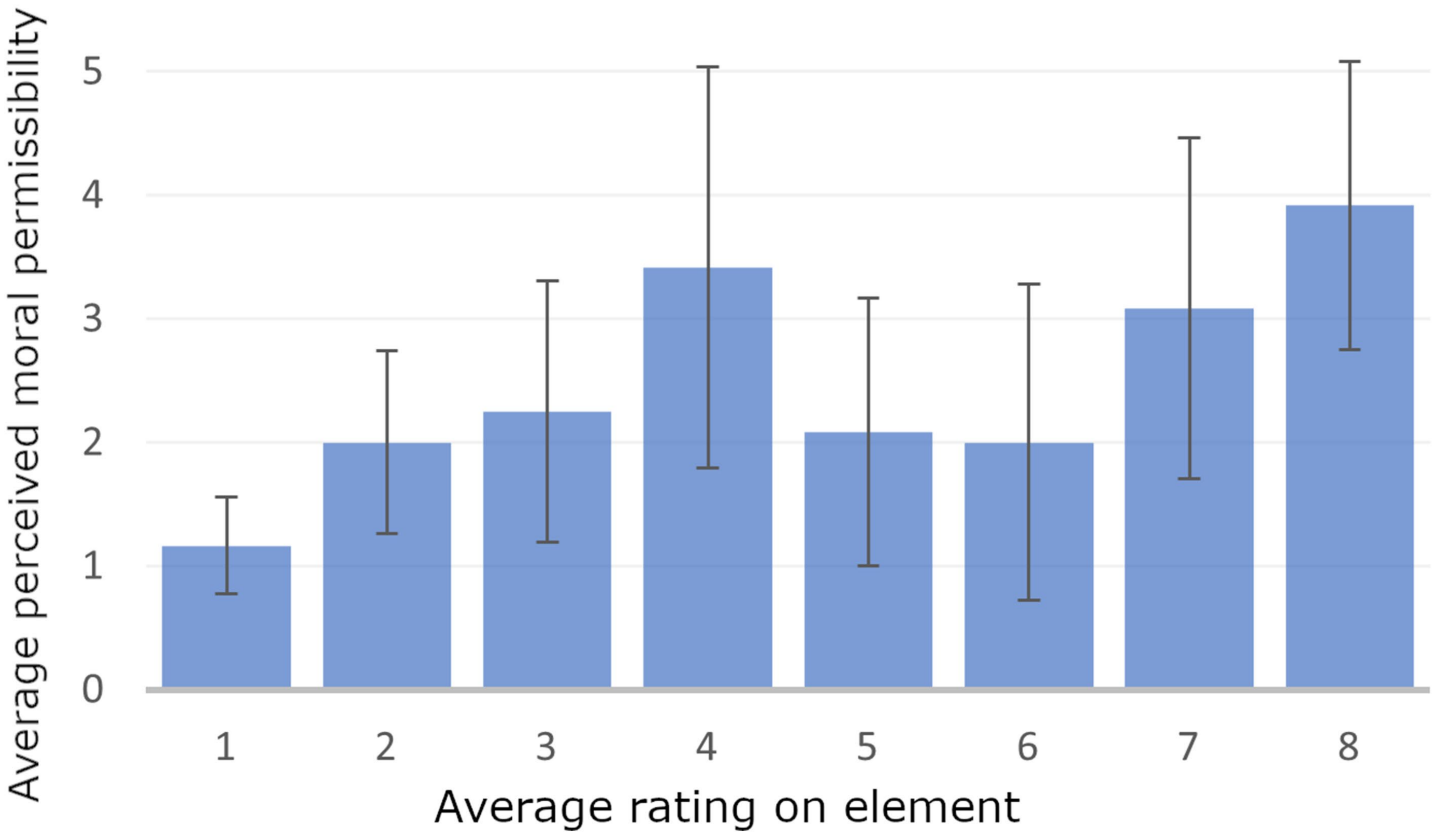
Chart showing the average ratings of the 8 elements (on the x-axis) on construct “moral wrongness” (on the y-axis), with a scale from 1 (morally permissible) to 5 (morally wrong). The chart also includes error bars representing the standard deviation.

As illustrated in Figure 3, participants generally perceived the Cogwar agent (element 8) as the most morally wrong, with the Anti-personnel mini drone (element 4) following closely behind. The data also indicates a high standard deviation for each construct, suggesting a wide variation in participants’ opinions.

Given the extensive data provided by participants on their personal constructs and their element-ratings according to these constructs, it might be tempting to connect them and identify which constructs influence judgments of moral wrongness. However, caution is needed when interpreting RGT data in this manner, as emphasized by Hassenzahl and Wessler (2000). Correlations between constructs would be based on limited data, with only 8 elements being rated, making it difficult to draw meaningful conclusions. Additionally, these correlations may not indicate causation. During the semi-structured interview that followed the repgrid data extraction phase, participants provided valuable insights into their motivations. They noted that they often needed more context to make well-founded assessments of moral permissibility, such as understanding how an algorithm is tested and its precise use conditions, which contributed to some of the disagreements in their evaluations. However, other factors also played a role. Some participants believed that the low reliability of the anti-personnel drone’s facial recognition algorithm would make it highly immoral to use, while others saw no fundamental difference between the anti-radar loitering munition and the anti-personnel drone. Additionally, some argued that the team-like interaction between the LLM-robot and the Teammate enhanced moral permissibility by keeping humans closely involved in the decision-making process. In contrast, others felt that these systems allowed overly broad instructions to the robot, potentially obscuring their immoral nature by permitting loosely defined interactions. Most people agreed that the cogwar agent would be highly immoral as it targets large amounts of citizens, produces highly unpredictable outcomes and affects people’s thinking (“*you cannot mess with someone’s brain!*”).

These open discussions highlighted the complexity of the subject, the diversity of perspectives, and how easily participants could end up talking past each other. This strengthens our belief that the MHC debate needs a more solid foundation rooted in commonly understood concepts, which will be addressed in the next section.

### 4.2 Interpretation

Many of the 156 constructs generated by participants were semantically similar but labeled differently. To address this, we organized them into categories called common constructs. This categorization was based on qualitative analysis of construct labels, associated values, and interview discussions, and required extensive manual effort. We conducted several rounds of analysis: one researcher proposed an initial grouping, which another then refined. This iterative process continued until we reached consensus. The results of this process are presented in Table 2.

**Table 2:** Common constructs resulting from grouping similar constructs that were mentioned by at least two interview participants (STS = Socio-technical System).

Common construct	Left pole	Right pole	Times mentioned	AvgStDev	STS component
Autonomy	Low autonomy	High autonomy	16	1.3	Technology
Destructiveness	Non-kinetic effects	Kinetic effects	14	1	Context
Timing of Control	Continuous control	Set and forget	12	0.7	Interaction
Embodiment	Virtual embodiment	Physical embodiment	11	0.6	Technology
Trust	Hard to develop trust	Easy to develop trust	7	1.4	Human
Human involvement	Human in the loop	Human out the loop	6	0.8	Interaction
Military status	Military	Civilian	6	0.9	Organization
Situation Awareness	Human requires high SA	Human requires low SA	6	1.1	Human
Operator-System Proximity	Close distance operator system	Far distance operator system	6	0.7	Interaction
Command Abstraction	Very precise instructions	Open instructions	6	1	Interaction
Lethality	Non-lethal effect	Lethal effect	4	0.5	Context
Moral Sensitivity	Morally insensitive	Morally sensitive	4	1.4	Context
Training	Limited training needed	Much training needed	3	1.4	Human
Team membership	Part of team	Not part of team	3	2.1	Interaction
Regulation	Low regulated	Highly regulated	3	0.6	Organization
Predictability of environment	Predictable environment	Unpredictable environment	2	0.7	Context
Abortability	Always abortable	Very limited abort window	2	0.6	Interaction

The table above presents the common constructs that emerged from grouping similar constructs mentioned at least two times by interview participants. The researchers determined the names of the common constructs and their corresponding poles, closely aligning them with how the participants referred or named those constructs. The list is ordered by the number of times the construct was mentioned by a participant (see the column “Times mentioned”). As revealed by the table, the constructs *Autonomy*, *Destructiveness*, *Timing of control*, *Embodiment* were raised most frequently by the participants. The fact that some common constructs were mentioned more times than there are participants can be attributed to some participants mentioning the same construct multiple times using different phrasing. All data were included in the analysis, and no data points were excluded as *outliers*.

When determining poles for the common constructs, we faced the challenge of some participants using reversed poles for the same construct. To address this, we corrected for these reversals and adjusted the element ratings accordingly, enabling us to perform correct comparisons such as averaging ratings and calculating standard deviations as discussed below.

The column AvgStDev of a common construct (CC) was calculated by:

$$\text{AvgStDev}(\mathrm{CC})=\frac{1}{n}\sum_{i=1}^{n}\sigma_{\left\{R(c,e_{i})|c\;\text{maps}\;\mathrm{on}\;\mathrm{CC}\right\}}$$

Where,

-
R(c,e) is the rating of construct c for element e (in our case, R(c,e)∈{1,2,3,4,5} ).
-
n are the number of elements (8 in our case).

This formula describes the average of the 8 elements’ standard deviations over the ratings of all constructs that map onto the common construct. Intuitively, the AvgStDev reflects the level of disagreement among participants about the meaning of a common construct. A low value indicates that participants gave similar ratings, suggesting relative agreement. A high value means that participants provided varying ratings, indicating disagreement on the construct’s meaning and its application to the elements. As revealed by the table, the constructs *Team membership*, *Trust, Moral sensitivity, Training* were used in ways that reflect disagreement.

The final column in the table shows the socio-technical system (STS) component, indicating that participants evenly distributed their constructs across all components of the socio-technical system, as instructed.

Out of the 156 elicited constructs, only 111 were grouped into one of the common constructs. The remaining 45 constructs fall into two categories. The first category includes *unique constructs*, which were mentioned by only one participant. In our study, there were 26 such constructs. These are not presented in Table 1, but interesting examples include *airborne/land-based*, *level of AI sophistication*, *established doctrine/unknown doctrine*. The second category includes constructs that were set aside because their meanings were unclear upon review, and we believed the interviewee had likely used a more suitable term elsewhere in their response. This phenomenon can be explained in interviewing theory by the concept of *laddering* (Curtis et al., 2008), where the interviewee gradually refines their answers through a series of successive clarifications, each becoming more specific over time.

The number of unique concepts (26) compared to common constructs (17) (i.e., much more uniqueness than sharedness in concepts to describe the same elements), says something about the complexity of the domain and the way in which the community has (not) established a shared communication vocabulary. However, using this ratio as a measure overlooks the fact that some common constructs are more frequently mentioned than others. For this purpose, we propose to apply set similarity comparisons. One option is the multi-set Jaccard Index (Costa, 2021), which is defined as $\mathrm{MSJ}(C_{1},C_{2},\ldots C_{n})=\frac{|C_{1}\cap C_{2}\cap \ldots \cap C_{n}|}{|C_{1}\cup C_{2}\cup \ldots \cup C_{n}|}$ , where $C_{i}$ denotes the construct set used by participant i adjusted to common construct names if applicable. Applying this formula to the construct sets raised by the 12 participants used in this study, we obtain a Jaccard Index of 0. This is because there is no common construct that was used by all participants, which results in the denominator of the Jaccard Index being 0. Since a value of 0 does not fairly represent the degree of similarity between the construct sets, we propose using an alternative measure known as the Average Pairwise Jaccard Index, which is defined as $\mathrm{APJ}(C_{1},C_{2},\ldots C_{n})=\frac{1}{\left(\begin{array}{c} n\\ 2 \end{array}\right)}\sum_{1\leq i<j\leq n}\mathrm{MSJ}(C_{i},C_{j})$ . This value would be 0 if none of the participants’ construct sets had any overlap. The greater the overlap, the higher this value would be. It would be 1 if all participants’ construct sets were exactly the same. Using a Python script, we computed the APJ for our dataset, and obtained a Pairwise Jaccard Index value of 0.1755. This low value suggests that there is limited overlap in the constructs raised by the participants. Since, to the best of our knowledge, no other study has applied this measure to repertory grid studies, we cannot determine if this value is unusually low compared to other domains.

**Figure 4:**
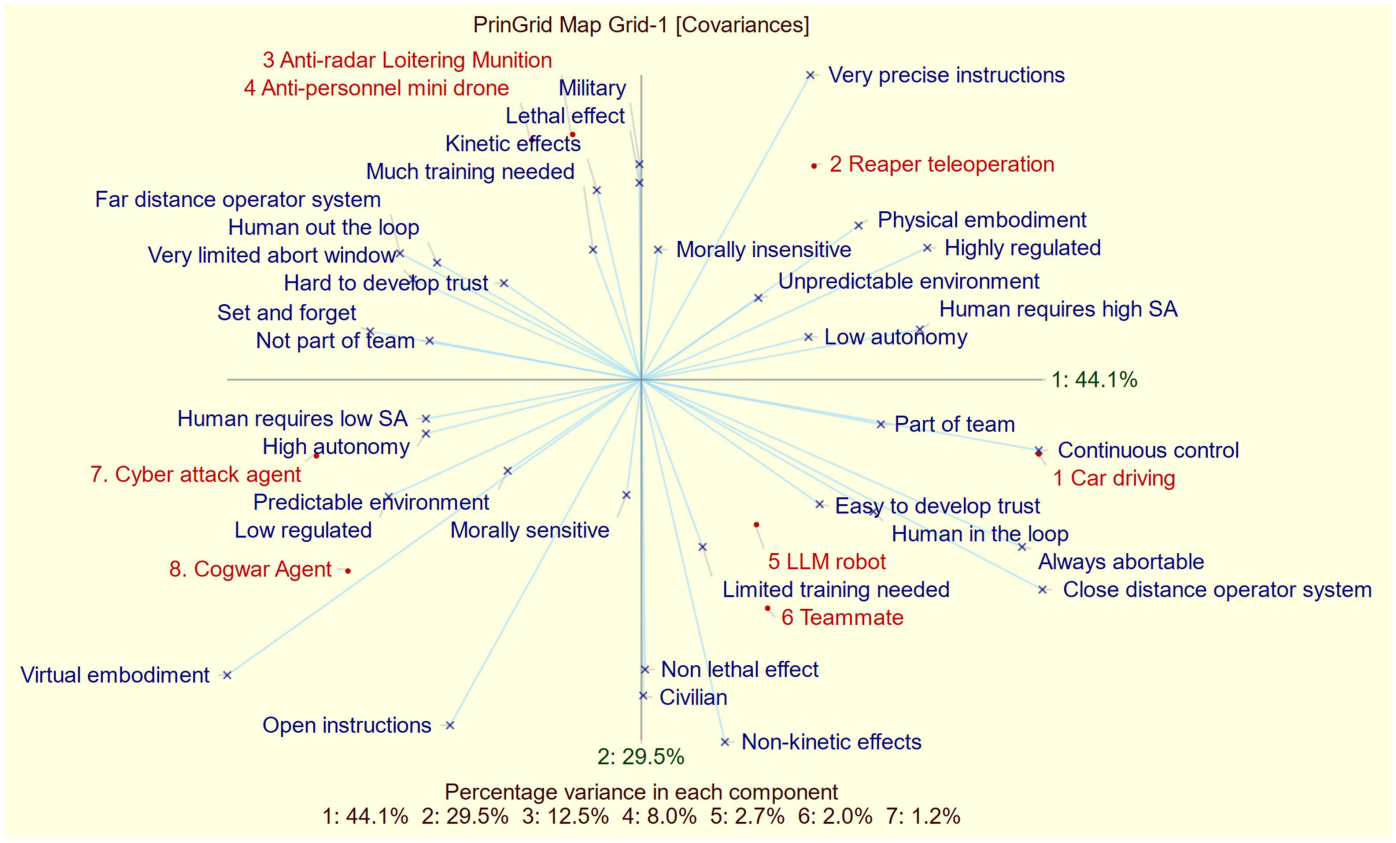
Principal component analysis of all common constructs.

Finally, we present the principal component analysis derived from all common constructs, using average element ratings from each construct that maps into it (Figure 4).

Just like the PCA of all data (plotted in Figure 2), the first two components account for most of the variance (i.e., 73.6%). This means that the two-dimensional plot gives a good impression on which elements were rated similar. Moreover, since this plot includes significantly fewer constructs compared to the one in Figure 2, it allows for a clearer interpretation of the constructs (represented by blue lines) and how they help differentiate between elements. To do this, plot the elements perpendicularly on the construct line and assess the resulting distance between the plotted elements. The greater the distance, the better this construct is suited in explaining the difference between the elements. For instance, if we want to compare element 8 (Cogwar agent) with element 2 (Reaper teleoperation), we would plot both elements perpendicularly on the line representing the construct “virtual embodiment – physical embodiment.” The resulting points would be noticeably distant from each other, meaning that the *embodiment* construct is helpful in explaining the difference between a cogwar agent and reaper tele-operation system. If we would plot these elements perpendicularly to the line labeled “Close Distance Operator System – Far Distance Operator System,” we would find that the resulting points are close to one another. This indicates that the construct is not very useful to distinguish between these elements. Keep in mind that this method is not perfect, as the-two dimensional plot only accounts for 73.6% of the variance.

### 4.3 Application

In the interpretation phase, we analyzed the language that experts use when discussing these systems and how they conceptualize them through that language. To make the findings more relevant to system design, we interpreted the results in terms of the specific requirements they allow us to define. For this purpose, we distinguish between the following types of requirements:

- Functional requirements: These requirements stem directly from the problem that needs to be solved or the goals of the stakeholders.
- Design requirements: These are requirements that the engineer sets based on specific design decisions and how the solution should be implemented.
- Derived requirements: Which arise as a natural consequence of the design choices. These are often not explicitly stated at the outset but become necessary as the design is developed. When fully developed, they may solidify into design principles.

**Figure 5:**
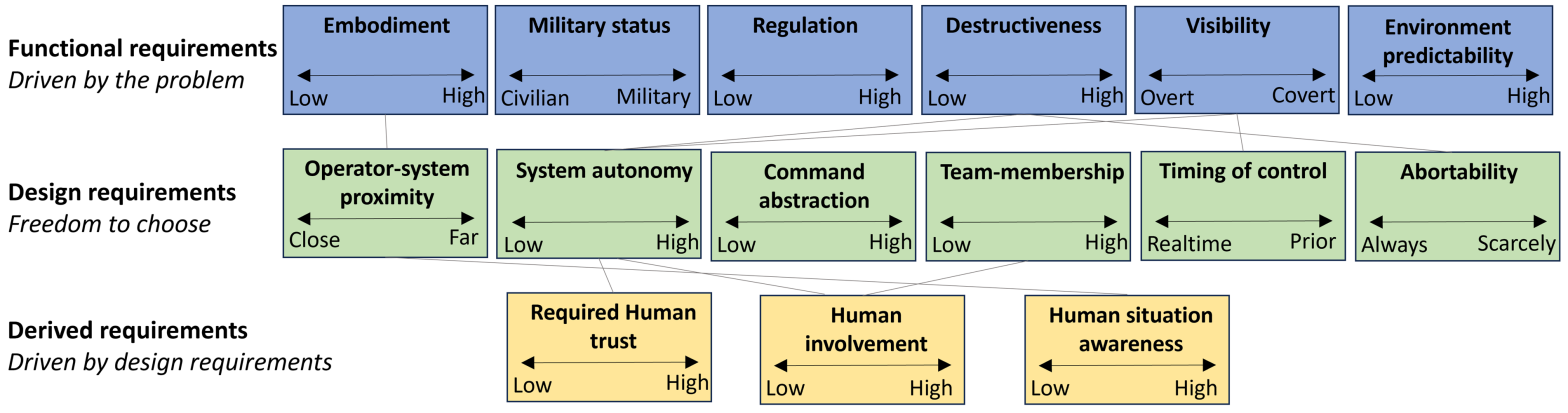
Basic design map for MHC extracted from the repertory grid data.

Through a manual classification effort by the research team, we categorized all common constructs into these three groups. Some requirements could fit multiple categories, depending on interpretation. Since distinctions in design are not always clear-cut, the research team made a judgment call. This resulted in 6 functional requirements, 6 design requirements, and 3 derived requirements (as visualized in Figure 5).

Because the design requirements are most relevant for system design, we will inspect them closer below by plotting their average values in a parallel coordinates plot.

**Figure 6:**
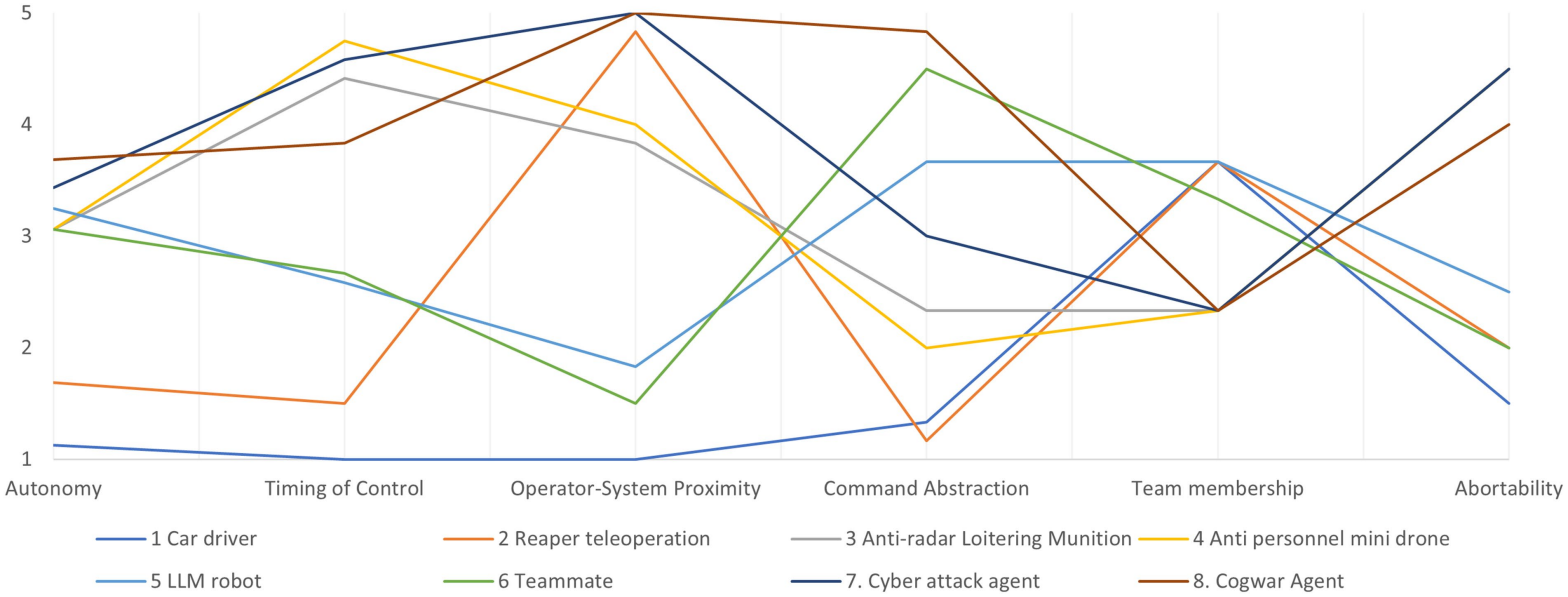
The common design requirements plotted in a parallel coordinates plot.

The plot in Figure 6 shows significant variation in elements across the dimensions defined by the design requirements, indicating substantial design freedom. This is expected to some extent, as participants were asked to generate constructs that highlight both differences and similarities. However, it also reveals that system differences manifest in the design space where engineers have options. Designers should be made aware of these choices to better support MHC. To enhance the design process using these abstract insights, various tools, such as *morphological charts* (Smith et al., 2012) and *abstraction hierarchies* (Vicente, 1999), can be utilized. Without delving deeply into these methods, we propose a simplified version of a design map inspired by them.

The figure above depicts layers corresponding to the three types of high level requirements discussed earlier, with lines between the layers representing dependencies. For instance, the level of *Visibility* influences the amount of *System Autonomy* as covert operations require minimizing radio signal transmission. Similarly, *System Autonomy* affects the level of *Human situation awareness* as highly autonomous systems do not require the human operator’s real-time involvement.

## 5 Limitations of the study

RGT strikes a balance between quantitative and qualitative data analysis. This can be seen as a strength, but others may consider this a weakness of the method. It places limits on the generalizability of findings as does any study with a relatively low number of participants does. Another limitation, which is inevitable, is that different people use different words to describe the same thing, or they use similar words, but ascribe a different meaning to them. This required the researchers to manually group construct labels that they thought had similar meanings, in order to make comparable common constructs. This means that some constructs may have been categorized in a way that the participants did not have in mind. Furthermore, we interviewed experts from different fields of research, but the individuals that we picked may hold strongly deviating views on the topic relative to the peers in their field. This is a risk with any expert interview. Recommendations to enhance the robustness of the findings is to add surveys, depth interviews, or comparative case studies. Another recommendation is to use a variety of techniques for data analysis, including automated text analyzers that may provide good quality outcomes given the advancements in large language models and AI.

## 6 Conclusion

The difficulty in establishing a multidisciplinary dialog on MHC hampers progress in establishing responsible implementation of AI in the military. This complexity underscores the need for a shared understanding and common vocabulary to bridge the gap between the various disciplines involved. This paper proposes the use of the RGT to arrive at a shared design space of MHC.

The two primary objectives of this study were: first, to explore how experts from various disciplines perceive MHC using RGT; and second, to propose a method for using these insights to foster mutual understanding and provide a practical framework for operationalizing MHC across different stakeholders.

The analysis of our interviews revealed that experts use highly different concepts and terms to understand MHC-related vignettes. However, we can identify a common vocabulary. We have demonstrated that one application of this common vocabulary is the creation of a design map, which illustrates the different options available to designers for incorporating MHC into their systems.

Regarding the RGT itself, participants reported that the RGT process was generally engaging and inspired them to think critically about the complexities of MHC, yielding a variety of constructs that reflect diverse perspectives on control, ethics, and system design.

This study offers several contributions compared to earlier design studies that explored the use of RGT for design problems. We proposed the clustering of multiple grids to identify shared constructs, which allowed us to quantify the level of consensus. This was measured using average standard deviation for individual constructs and Pairwise Jaccard distance for all constructs. Additionally, we demonstrated how the transition to a design map can be facilitated by a parallel coordinates chart. The resulting design map for MHC, based on these shared constructs, provides insight into the design space and supports engineers in decision-making when working on MHC. It serves as a practical guide with design patterns to enable a variety of options for MHC outcomes in design of human-AI systems.

Our study demonstrated that the RGT is an effective tool for addressing ethically complex design challenges like MHC, which require interdisciplinary collaboration. It enables in-depth exploration of expert knowledge while striking a balance between open-ended interviews, which foster free-flowing discussion but lack statistical analysis, and structured questionnaires, which allow for statistical analysis but limit conversation to predefined ideas. However, we have argued that applying RGT effectively for this purpose also requires a time-intensive manual analysis process, which is not supported by currently available RGT tools.

Future research could focus on enhancing methods for eliciting and analyzing construct sets that start with an established vocabulary. For instance, this could involve refining the construct set identified in this study through additional iterations. Further exploration could also examine the wider application of RGT in other aspects of responsible AI design.

## Data Availability

The datasets presented in this article are not readily available because to preserve privacy of interview participants, the data will not be made fully available. Requests to access the datasets should be directed to jurriaan.vandiggelen@tno.nl.

## Footnotes

^1^ https://www.nato.int/cps/en/natohq/official_texts_227237.htm

^2^ https://www.esd.whs.mil/portals/54/documents/dd/issuances/dodd/300009p.pdf

^3^ https://artificialintelligenceact.eu/article/3/

^4^ https://www.oecd.org/content/dam/oecd/en/publications/reports/2024/03/explanatory-memorandum-on-the-updated-oecd-definition-of-an-ai-system_3c815e51/623da898-en.pdf

^5^ https://youtu.be/O-2tpwW0kmU?si=nwMr8F9OPxHCf7OY

^6^ https://youtu.be/Vq_DcZ_xc_E?si=uWMUIY1rNID0sKyp

^7^ https://youtu.be/HaZZ499P8oo?si=qK6a0_huYEkHaiF3&t=37

^8^ https://www.kaspersky.com/resource-center/definitions/what-is-stuxnet

^9^ https://youtu.be/xoVJKj8lcNQ?si=GeUIvQiaPURh-bDB&t=2626

^10^In this paper, this example is used to briefly illustrate the procedure of RGT. For a thorough treatment of the repertory grid interview technique (see Tan and Hunter, 2002).

^11^ https://cspages.ucalgary.ca/~gaines/repplus/
